# Surveillance and identification of clusters of healthcare workers with coronavirus disease 2019 (COVID-19): Multidimensional interventions at a comprehensive cancer center

**DOI:** 10.1017/ice.2020.1315

**Published:** 2020-11-13

**Authors:** Ella J. Ariza-Heredia, Elizabeth Frenzel, Sherry Cantu, Mary Carlson, Georgia Thomas, Fareed Khawaja, Issam I. Raad, Roy F. Chemaly

**Affiliations:** Department of Infectious Diseases, Infection Control and Employee Health, The University of Texas MD Anderson Cancer Center, Houston, Texas

## Abstract

**Background:**

Cases of novel coronavirus disease 2019 (COVID-19) were first reported in Wuhan, China, in December 2019. In this report, we describe 3 clusters of COVID-19 infections among healthcare workers (HCWs), not associated with patient exposure, and the interventions undertaken to halt ongoing exposure and transmission at our cancer center.

**Methods:**

A cluster of cases was defined as 2 or more cases of severe acute respiratory coronavirus virus 2 (SARS-CoV-2)–positive COVID-19 among HCWs who work in the same unit area at the same time. Cases were identified by real-time reverse transcription polymerase chain reaction testing. Contact tracing, facility observations, and infection prevention assessments were performed to investigate the 3 clusters between March 1 and April 30, 2020, with subsequent implementation of containment strategies.

**Results:**

The initial cluster involved HCWs from an ancillary services unit, with contacts traced back to a gathering in a break room in which 1 employee was symptomatic, although not yet diagnosed with COVID-19, with subsequent transmission to 7 employees. The second cluster involved 4 employees and was community related. The third cluster involved only 2 employees with possible transmission while working in the same office at the same time. A step-up approach was implemented to control the spread of infection among employees, including universal masking, enhanced cleaning, increase awareness, and surveillance testing. No nosocomial transmission to patients transpired.

**Conclusions:**

To our knowledge, this is the first report of a hospital-based cluster of COVID-19 infections among HCWs in a cancer hospital describing our steps to mitigate further transmission.

In December 2019, cases of novel coronavirus disease 2019 (COVID-19), caused by the recently named severe acute respiratory syndrome coronavirus 2 (SARS-CoV-2), were first reported in Wuhan City of Hubei Province of China.^1^ The virus has spread all around the world causing devastation and unprecedented changes in the medical field, global economy, and infection control practices.^2^ On March 11, 2020, the World Health Organization (WHO) declared that the scientific criteria for a coronavirus pandemic had been met, with >4.9 million confirmed COVID-19 cases, 327,738 deaths, and 216 territories with known cases around the world as of May 22, 2020.^3^

SARS-CoV-2 spreads by human-to-human transmission via droplet exposure and contact transfer,^4^ causing infection that ranges from asymptomatic to severe such as acute respiratory distress syndrome (ARDS) and death.^2^ Risk factors associated with worse outcomes for patients with COVID-19 include older age, history of hypertension,^5^ acute and chronic kidney disease,^6^ and diabetes.^7^ Because patients receiving oncological care typically have continuous contact with healthcare settings and because they are known to be at higher risk of complications from respiratory viral infection,^8^ our institution has accentuated best practices from the beginning of the pandemic to minimize patient and healthcare worker (HCW) exposure to SARS-CoV-2 while continuing to provide necessary care.

Moreover, SARS-CoV-2 has affected HCWs and medical facilities in an unprecedented way, and prompt strategies are of paramount importance to ensure the containment of the disease among staff and patients, which has been challenged by the rapidly changing guidance from national and international governmental and healthcare organizations.^9^ In this report, we present 3 clusters of HCWs with COVID-19 and discuss the interventions undertaken to halt ongoing exposure and transmission at our cancer center. Here, we highlight important practices that could be useful for the overall medical community as SARS-CoV-2 continues to threaten society and healthcare facilities.

## Setting

The University of Texas MD Anderson Cancer Center is a National Cancer Institute–designated comprehensive cancer center in Houston, Texas, with ~680 hospital beds and ~22,000 employees.

## Case definition

A cluster of cases was defined as 2 or more cases of SARS-CoV-2–positive COVID-19 among HCWs who work in the same unit area at overlapping times. The identification of cases was carried out by testing symptomatic and asymptomatic HCWs with a reverse transcription polymerase chain reaction (RT-PCR) assay specific for SARS-CoV-2. Contact tracing, facility observations, infection prevention assessments, employees questionnaire, and testing of symptomatic and asymptomatic HCWs were performed to investigate the ongoing transmission of SARS-CoV-19 and to identify potential sources of COVID-19. This study was approved by our institutional Quality Improvement Assessment Board as a quality improvement project.

## Description of Clusters

### Cluster 1

On March 27, 2020, ~15 ancillary services staff congregated in a small break room (~21 m^2^ or 249 ft^2^) for a celebration despite the implementation 10 days earlier of a social distancing policy and a policy that limited gatherings to up to 5 HCWs. The HCWs did not wear face masks at the event because masking was adopted as an institutional policy for employees who interact with patients on March 28, 2020, and for all personnel on April 1, 2020. On March 30, 2 of the employees who participated in the gathering became ill with respiratory symptoms including cough, shortness of breath, and fever, and on April 1, they tested positive for SARS-CoV-2. After further inquiry, a third employee (probably the “index case”) who tested positive on April 9, reported being symptomatic with mild upper-respiratory symptoms, including sore throat, and runny nose at the time of the gathering on March 27, but these HCWs did not convey these symptoms to the employee health department until April 7.

**Fig. 1.**
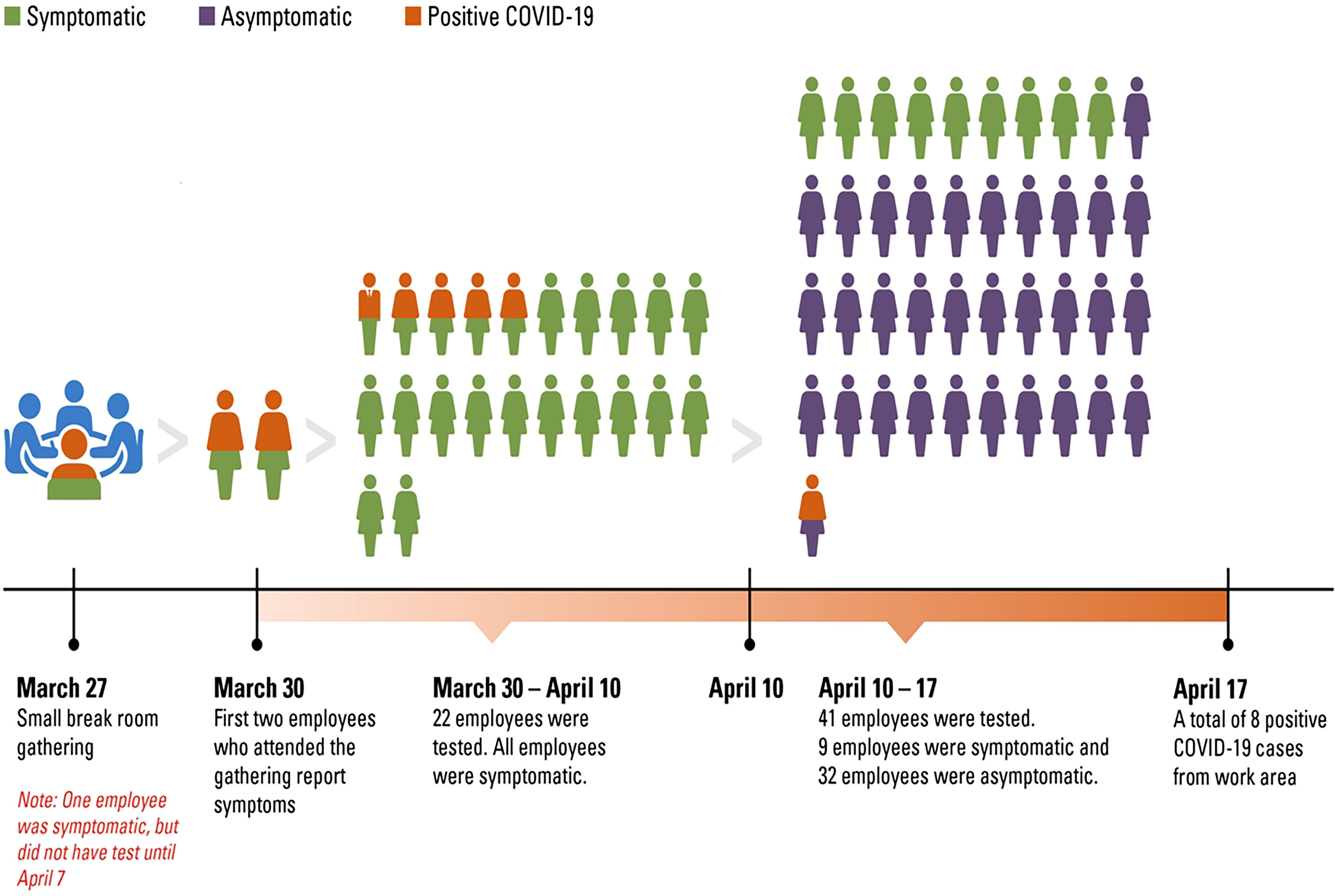
Sketch showing timeline of cluster #1.

After the first 2 employees tested positive, the information was shared with the manager of the area. Immediately, communication was circulated to all staff in the ancillary services unit (with anonymous and deidentified information to protect the confidentiality of the COVID-19 positive employees) to encourage heightened awareness, self-monitoring for respiratory symptoms, and subsequent testing for SARS-CoV-2 (Fig. 1). At this juncture, multiple interventions were promptly implemented:

1. Further reinforcement through internal communications encouraged employees to report any respiratory symptoms and to follow-up with testing if symptoms-based criteria were met.
2. Enhanced terminal cleaning of the ancillary services unit by housekeeping personnel was requested.
3. Reinforcement and monitoring of masking at all time for all employees was implemented.
4. Social distancing was emphasized. The break room was closed until further notice and gathering in any other common areas was prohibited.
5. Asymptomatic employees in the same area were tested.
6. All positive COVID-19 HCWs were isolated for at least 10 days from the date of symptom onset and until improvement in symptoms for at least 3 days.

Briefly, between March 30 and April 10, 22 employees with symptoms varying from body aches, runny nose, shortness of breath, and fever were tested, and 7 (31%) were positive for COVID-19 from the same working area of the first 3 employees (Table 1). Upon interviews, none of the employees reported recent travel or outside contact with known SARS-CoV-2–positive or -suspected individuals. After April 10, testing of asymptomatic employees in the same area was initiated. Overall, 32 asymptomatic employees were tested and 1 (2%) was positive for SARS-CoV-2 (Fig. 1). In summary, all employees from the area (n = 63) were traced and tested, and 8 were positive for SARS-CoV-2, including 1 asymptomatic employee (positivity rate, 13%). After the last asymptomatic positive COVID-19 case was diagnosed on April 13, no additional positive cases were identified or detected in that area. Importantly, there were no positive cases of COVID-19 among patients who visited this area during their care at our institution and up to 14 days from the last diagnosed employee.

**Table 1.** Description of Cases of Cluster 1

Employee No.	Onset of Illness	Positive SARS-CoV-2 Test	Employee Area	Last Day of Work
1	3/30/2020	4/1/2020	Ancillary services unit	3/31/2020
2	3/31/2020	4/5/2020	Ancillary services unit	4/1/2020
3	3/30/2020	4/1/2020	Ancillary services unit	4/1/2020
4	4/2/2020	4/7/2020	Ancillary services unit	4/2/2020
5	3/27/2020	4/9/2020	Ancillary services unit	4/7/2020
6	4/7/2020	4/9/2020	Ancillary services unit	4/7/2020
7	3/30/2020	4/10/2020	Ancillary services unit	4/3/2020
8	Asymptomatic testing	4/13/2020	Ancillary services unit	4/15/2020

### Cluster 2

Based on our surveillance by evaluating every positive HCW, we identified 4 employees who worked on the same inpatient floor but in different units who tested positive for SARS-CoV-2 between April 3 and April 6, 2020. All 4 HCWs experienced respiratory symptoms before testing and were quarantined at home according to our institutional policies once symptoms were reported. After investigation, we determined that the employees did not work together during that period and did not have contact with known COVID-19 patients in the hospital. All employees wore a mask while in contact with patients, and no nosocomial transmission was detected for at least 4 weeks from cluster identification. Community exposure and transmission were suspected.

### Cluster 3

In cluster 3, 2 coworkers who shared the same office were diagnosed with COVID-19 on April 17 and April 21, respectively. One of these employees had symptom onset on April 17, and after evaluation at an outside facility, SARS-CoV-2 testing was not done. After symptoms were reported to our employee health department on April 21, a SARS-CoV-2 test was positive. The last day on campus for that employee was April 17. The second employee who shared the same office and did not have symptoms underwent testing on April 21 as part of our contact tracing (last day at work), which was positive. Their work areas are ~2 m apart, and although both reported wearing masks at work, they occasionally pulled them off when talking on the phone. Neither employee had had patient contact.

## Employee health and infection control assessments

At the beginning of the COVID-19pandemic in the United States, several policies related to COVID-19 and employees were established at our institution. We implemented multidimensional strategies to prevent exposure and transmission of SARS CoV-2 in our healthcare setting:

1. Reduced the workforce access to the institution
2. Enabled remote work from home with information technology support
3. Reduced by half of inpatient and outpatient care activities at our institution by April 2020.
4. Initiated entry-point screening for respiratory symptoms and travel history for patients and employees with separate entry-point access
5. Gradual reduced the number of visitors accompanying patients from 2 to none per patient.
6. Required personnel protection equipment for all employees, for example, to wear face masks in clinical or nonclinical areas of the institution at all times (as of April 1, 2020)
7. Implemented constant communications for all employees to heighten awareness, to perform self-monitoring, to not report to work when ill, to use and preserve personnel protective equipment, and other strategies.
8. Enhanced daily and terminal cleaning in clinical and common areas including team rooms and breakrooms with focus on high-touch surface areas such as elevators buttons, door knobs, and keyboards
9. Implemented social distancing including limiting in-person meetings and individuals in elevators to 5 or less
10. Provided access to SARS-CoV-2 testing for symptomatic or asymptomatic employees.
11. Monitored compliance with best practices such as universal masking, social distancing in work rooms, and hand hygiene, and addressing noncompliance instantaneously
12. Promoted self-reporting by employees of any COVID-19–related safety concerns via e-mail, an online reporting system, or by telephone through a COVID-19 hotline.
13. Based return-to-work policies for SARS-CoV-2–positive employees on the Centers for Disease Control and Prevention (CDC) recommendations. We chose the “symptom or time-based strategy” for return-to-work which entailed returning to work at least 10 days after symptoms first appeared with resolution of fever for 72 hours without the use of fever-reducing medications, and respiratory symptoms must have returned to baseline. The “time-based strategy,” which is utilized for employees with laboratory-confirmed SARS-CoV-2 without symptoms, is 10 days since the date of the positive test. Employees who are isolated for any reason must receive clearance from the employee health department to return to work.

## Discussion

Continued prioritization of SARS-CoV-2 transmission prevention in HCWs is crucial to reducing the impact of the COVID-19 pandemic on vulnerable patients and healthcare systems until an effective vaccine and treatments are developed. Herein, we report the investigation and prompt mitigation of 3 clusters of HCWs with COVID-19 at a large cancer center. Clusters 1 and 3 were associated with the work environment, whereas cluster 2 was most probably not. For cluster 1, 7 employees became ill with respiratory symptoms within 3 days of attending a small gathering in a break room and the eighth employee was asymptomatic and tested positive as part of the active investigation and surveillance testing. The index case was the person who was symptomatic prior to the gathering and who had delayed testing. For cluster 3, transmission occurred in a close environment between 2 employees who share the same office where masking was not practiced at all time.

**Fig. 2.**
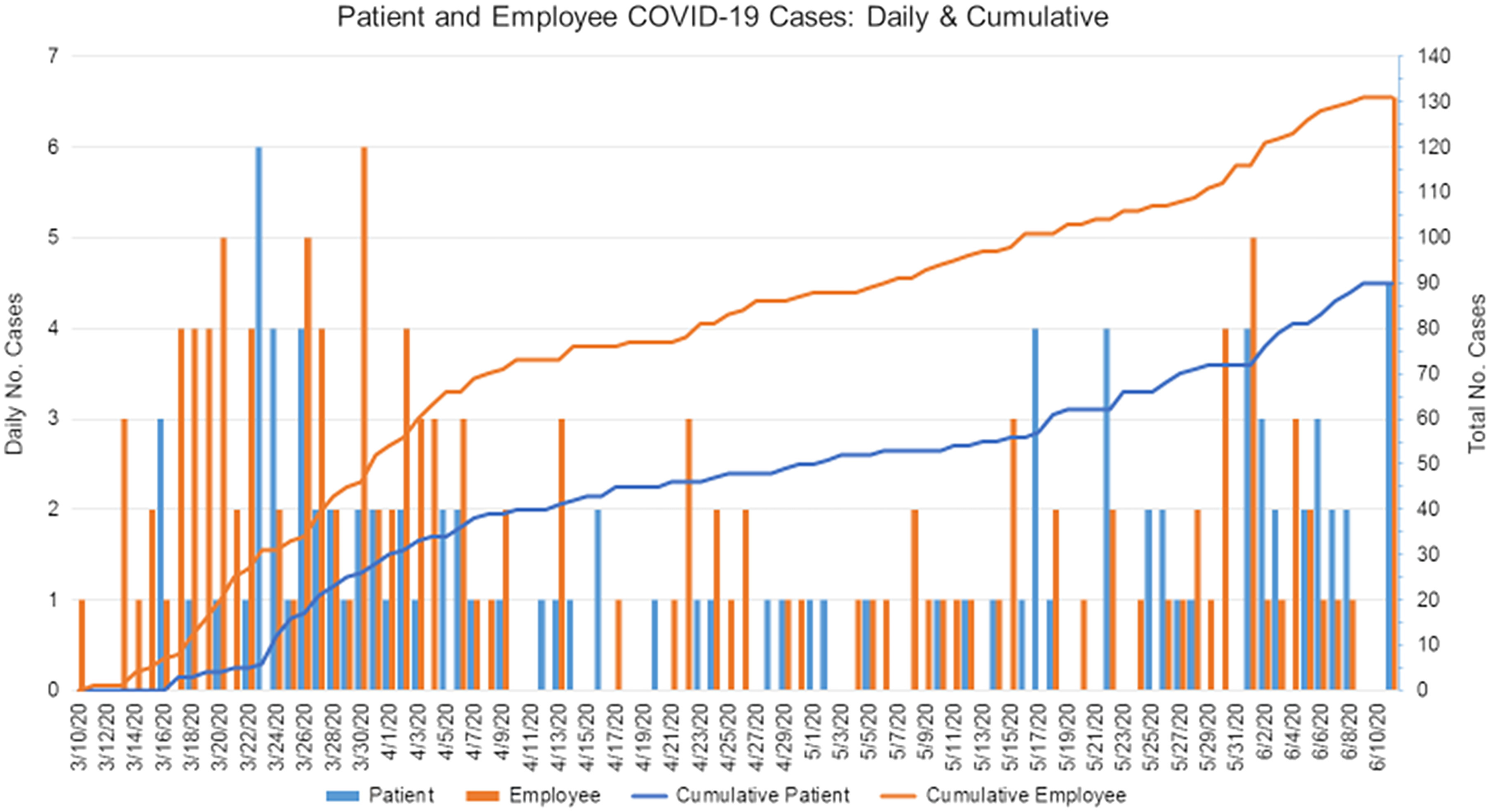
Summary of the cumulative incidence of confirmed COVID-19 cases among our employees and patients.

Multiple studies have shown that HCWs make up a significant proportion of COVID-19 cases. In the United States, 19% of the reported COVID-19 cases to the CDC were HCWs.^28^ In a single-center case series in China, up to 29% of COVID-19 were HCWs.^29^ HCWs also frequently interact with vulnerable, at-risk populations such as patients with cancer and could transmit the disease unknowingly. Thus, strict measures such as appropriate personnel protective equipment and testing should be prioritized for HCWs who have frequent contact with infected individuals. Figure 2 depicts the cumulative incidence of confirmed employees and patients with COVID-19 at our institution as of June 10, 2020.

From these clusters of cases, we established the high likelihood of transmission of SARS-CoV-2 to >1 individual from indoor exposure during gatherings and, thus, the importance of limiting number of personnel present in confined areas and of social distancing.^9–11^ We suspect that social distancing in conjunction with universal face masks would have prevented this outbreak.^9–11^ In addition, and most importantly, a low threshold for suspecting and testing for COVID-19 in otherwise healthy individuals with minimal or mild respiratory symptoms is of utmost importance.

Around the world, continuous and vigorous efforts are ongoing to determine the best practices for limiting the spread of SARS-CoV-2 within communities and healthcare facilities, which have been at the epicenter of the pandemic. Respiratory viruses are spread by direct contact or by droplet transmission produced by infected individuals from coughing or sneezing, for example, and by direct contact with secretions followed by self-inoculation to the eyes, nose, and mouth. Thus, prevention measures have focused on using masks, washing hands, and enhanced cleaning of areas that could have been touched by an infected person.^10^ In general, large respiratory droplets (>5 μm) remain in the air for a short time and travel short distances; hence, maintaining at least 2 m between people is recommended to help prevent infection.^10,11^ Specifically, several studies on SARS-COV-2 have demonstrated not only droplet transmission of the virus^10,11^ but also a less common route of airborne transmission that translates into the virus remaining longer in the air and travelling >2 m away from the infected individual. Furthermore, a recent report from China on a cluster of COVID-19 cases in a restaurant demonstrated how droplet transmission can be affected by the direction of the air-conditioned ventilation, which can propagate droplets and increase transmissibility.^10,12^ Studies conducted during and after the SARS epidemic in 2003 suggested that airborne spread may have played an important role in the transmission of that disease, since some of these droplets could be aerosolized in some circumstances.^13–15^ To determine the pattern followed by SARS-CoV-2, and if similar to SARS, more data are needed as experts cannot agree about airborne potential to date.^16^ In addition, SARS-COV-2 may remain stable for days on different surfaces in the environment including plastic and stainless steel, copper, and cardboard.^19^ Whether these fomites translate into an efficient route of transmission among those who are not in direct contact with infected patients remains to be determined.^17^ In addition to the heterogeneity of the transmission pathways, other important aspects that affect the spread of a respiratory virus include the degree of infectivity of each individual, the lack of previous immunity, the risk of transmission by asymptomatic persons, and the number of contacts to whom each individual may be able to transmit the disease, as has been demonstrated in other rapidly spreading viral infections.^18–20^

Given the novelty of SARS-CoV-2 and the swiftness of the disease transmission, protocols and policies for infection control and prevention practices at the public health and hospital levels have been changing rapidly.^21,22^ Currently, preventing the spread of SARS-CoV-2 is the primary public health intervention.^23^ By February 25, 2020, China had reported 3,387 infected HCWs in Hubei alone, of whom at least 18 died, causing growing concern among HCWs. From previous studies of respiratory viral infections, it is known that face masks can provide protection for HCWs against respiratory illness, although it has been challenging to be able to clearly define their efficacy.^24^ Descriptions of clustered infections among hospital personnel have been published previously with other coronavirus infections, including MERS and SARS, for which the use of universal face masks and N95 masks were associated with decreased transmission when used appropriately.^18,25,26^ In addition, a cluster of COVID-19 cases from a restaurant in Wuhan, China, highlighted the potential risk of transmission in indoor spaces, where the use of masks and social distancing (of at least 2 m) may have provided a level of protection and possible reduction in transmission.^10,27^

As we prepare to resume full activities and reopen offices and clinics, the risk of droplet transmission, especially in indoor spaces in clinical and nonclinical areas, must be taken into account. A recent study by Liu et al evaluated the concentrations of SARS-CoV 2 in different areas of 2 hospitals in Wuhan, China.^30^ The study demonstrated that most of the sites (including ICU and patients rooms) have undetectable or very low concentrations of SARS-CoV-2 aerosol, except for (1) patient toilets, probably via suspended virus-laden aerosols, (2) the protective apparel removal rooms, probably by resuspension of virus-laden aerosol from the surface of HCW PPE while being removed, and (3) crowd-gathering sites where patients or other personnel frequently pass.

In the present report, we describe the successful containment of COVID-19 spread among HCWs at our center, although we faced many challenges such as timely reporting symptoms and testing and the lag time between identifying the clusters and implementing the mitigating strategies. In addition, many other HCWs tested positive for SARS-CoV-2 (Fig. 2), but their infections were deemed community acquired because most of them had had contact with a positive household member, had travelled domestically, or had attended a large gathering outside our institution. These clusters of cases occurred before the implementation of strict social distancing guidelines and universal masking. Because many other transmission-prevention measures were also implemented at our institution, we were not able to determine the distinct impact of universal masking in the workplace when additional measures are in place such as active surveillance, robust screening, and/or testing program as well as strict infection control practices such as hand hygiene.

Our report has several limitations. First, we did not conduct an experimental study to evaluate the droplet or airborne transmission routes or ventilation systems, which have been key in other investigations. Second, we did not obtain genotypic data on the SARS-CoV-2 samples from our HCWs, and lastly, some aspects of our case descriptions are subject to bias recollection from the employees, a limitation inherent in all survey studies. However, our report relates several educational points that we believe will be of great use by other medical centers.

In conclusion, a high level of alert and active surveillance are needed, especially in healthcare facilities, to early detect and prevent the spread of any respiratory viral infection, not only in in-patient care areas but also in areas with no direct patient contact. As we prepare to reopen activities and to prevent the spread of COVID-19 in healthcare facilities, special attention must be placed on gathering areas, food services areas, and break rooms on campus. In addition, universal masking, social distancing, contact tracing, appropriate testing capacity, and compliance with institutional policies are of utmost importance in the mitigating the spread of COVID-19.
